# The Tumor Suppressors p53, p63, and p73 Are Regulators of MicroRNA Processing Complex

**DOI:** 10.1371/journal.pone.0010615

**Published:** 2010-05-12

**Authors:** Lakshmanane Boominathan

**Affiliations:** Genome Discovery, Murungapakkam, India; Roswell Park Cancer Institute, United States of America

## Abstract

The tumor suppressors p53, p73, and p63 are known to function as transcription factors. They promote either growth arrest or apoptosis, depending upon the DNA damage. A number of microRNAs (miRNAs) have been shown to function as transcriptional targets of p53 and they appear to aid p53 in promoting growth arrest and apoptosis. However, the question of p53/p63/p73 regulating the miRNA processing complex has not been addressed in depth so far. Comparative/computational genomic analysis was performed using Target scan, Mami, and Diana software to identify miRNAs that regulate the miRNA processing complex. Here, I present evidence for the first time that the tumor suppressors p53, p63, and p73 function as both positive and negative regulators of the miRNA processing components. Curated p53-dependent miRNA expression data was used to identify p53-miRs that target the components of the miRNA-processing complex. This analysis suggests that most of the components (mRNAs' 3′UTR) of the miRNA processing complex are targeted by p53-miRs. Remarkably, this data revealed the conserved nature of p53-miRs in targeting a number of components of the miRNA processing complex. p53/p73/p63 appears to regulate the major components of the miRNA processing, such as Drosha-DGCR8, Dicer-TRBP2, and Argonaute proteins. In particular, p53/p73/p63 appears to regulate the processing of miRNAs, such as let-7, miR-200c, miR-143, miR-107, miR-16, miR-145, miR-134, miR-449a, miR-503, and miR-21. Interestingly, there seems to be a phenotypic similarity between p63*^−/−^* and dicer*^−/−^* mice, suggesting that p63 and dicer could regulate each other. In addition, p63, p73, and the DGCR8 proteins contain a conserved interaction domain. Further, promoters of a number of components of the miRNA processing machinery, including dicer and P2P-R, contain p53-REs, suggesting that they could be direct transcriptional targets of p63/p73/p53. Together, this study provides mechanistic insights into how p53, p63, and p73 regulate the components of the miRNA processing; and how p53, TA-p63, and TA-p73 regulated miRNAs inhibit tumorigenesis, EMT, metastasis, and cancer stem cell proliferation.

## Introduction

MicroRNAs (miRNAs/miRs) bind to 3′UTR/CDS of mRNAs and thereby they either promote their degradation or inhibit their translation. MiRNAs are processed by Microprocessors in the nucleus. Microprocessor consists of, namely, Drosha or RNASEN, an RNAse III endonuclease, and DGCR8, a double stranded RNA binding protein, The Drosha in complex with the DGCR8 processes (primary) pri-miRNA of several hundred base pairs in length into stem loop structure containing 60-70-nt (precursor) pre-miRNAs. Microprocessor also appears to consist of RNA helicases, such as, p68 and p72. Both these proteins have been shown to unwind double stranded stem loop containing pre-miRNAs, and thereby they enhance their processing. The pre-miRNAs are then transported out of the nucleus by RAN GTPase/Exportin5(Expo5). The pre-miRNAs present in the cytoplasm are processed by Dicer (an RNAse III endonuclease) containing complex (miRNA-generating complex). This complex consists of proteins, such as Dicer and TARBP2 (TAR binding protein 2). The Dicer in conjunction with the TARBP2 cleaves 60-70-nt pre-miRNAs into 21-22-nt mature miRNAs. This maturation process is aided by Argonaute endonucleases (Agos), such as EIFC1/Ago1 and EIFC2/Ago2, which bind to Dicer and TARBP2. The complex containing Dicer, TARBP2, Agos, and additional associated proteins is called as RNA-induced silencing complex. The Ago1/2 enhances the processing efficiency and cleavage of miRNAs. When there is a perfect match between miRNAs and mRNA sequences, the Ago1/2 promotes the degradation of mRNAs, while there is an imperfect match between miRNAs and mRNAs sequences, it promotes translational repression. Of interest, Dicer not only plays a role in the maturation of miRNAs, but also in siRNAs.

Considering miRNAs regulate one third of the genes present in the human genome, it is important to understand how they are regulated. MiRNAs, such as miR-34[a, b, c], miR-203, miR-29, let 7, miR-15, and miR-16, and their processing components have shown to be down regulated in multiple cancers. Ablation of miRNA processing enzymes, such as Dicer1, Drosha, DGCR8, and TARBP2, in cancer cells promotes more transformed phenotype and invasive tumors, suggesting that miRNA processing enzymes are required to control tumor- and metastasis-initiating events. Importantly, ∼50% of miRNAs are associated with fragile sites of chromosomes, suggesting that they could play a major in the control of cancer cell proliferation. In addition, miRNA components, such as DGCR8, XPO5, TNRC6B, Ago1, Ago 2, TARBP2, HSPCA, and SERBP1, have shown to be down regulated in lung cancer [1]. Interestingly, several miRNAs appear to target the expression of the tumor suppressor p53 related proteins, such as, p53, p63, and p73. p53 expression is inhibited by miR-125b [2], while p63 is inhibited by miR-302, miR-21, and miR-92 [3], [4], [5]. Based on these data, I have hypothesized that the tumor suppressors, such as p53, p63, and p73, could regulate the miRNA processing components.

## Results and Discussion

### p63 is a regulator of microRNA processing complex

The RNAse III ribonuclease, Dicer, has been shown to play a role in stem cell renewal. Conditional knockout of Dicer in mouse embryonic stem cells results in defective embryonic stem cell proliferation/differentiation, indicating that Dicer plays a significant role in the control of stem cell renewal [6]. Inhibition of Dicer expression has also been shown to down regulate the expression of stem cell factors, such as Oct4 [Pou5f1], Sox-2 and Nanog, indicating that its expression is required for the increased expression of self-renewal factors [7]. Likewise, the Microprocessor component DGCR8 appears to play a role in stem cell renewal and skin development [6], [7], [8]. In addition, a defect in the DGCR8 gene has been shown to associate with a syndrome, which is characterized by thymic hypoplasia, congenital heart defects, and facial abnormality [9]. Interestingly, a number of reports strongly suggest that p63 plays a major role in stem cell maintenance, skin development, thymic proliferation, and cardiac morphogenesis [8], [10], [11], [12], [13], [14], [15]. In addition, both TA-p63 and Dicer have been shown to play a critical role in protecting the germ line integrity of oocytes [16], [17], suggesting a phenotypic match between Dicer, DGCR8, and the p63 null mice.

**Figure 1:**
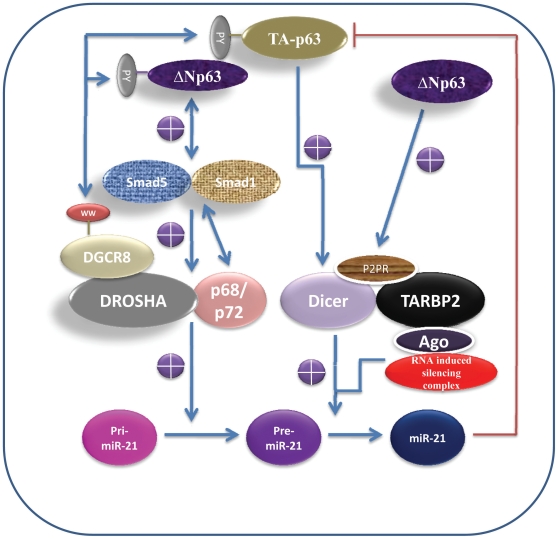
p63 is a regulator of microRNA-processing pathway.

It has previously been shown that WW domain-containing proteins interact with the PY domain-containing proteins. Remarkably, DGCR8 contains a WW domain [18], while p63 contains a PY domain [19], indicating a possibility of an interaction between the WW domain of DGCR8 and the PY domain of p63 [Figure 1]. Interestingly, it has been postulated that Drosha interacts with the WW-domain of DGCR8 through its PY domain [18]. Together, these data suggest that: a) p63 and DGCR8 proteins contain a conserved interaction domain; b) p63 could be part of the Microprocessor complex in the nucleus; and c) p63, by interacting with DGCR8, it could influence its ability to process pre-miRNAs.

Further, it has been shown that p63 null mice fail to develop hair follicles. However, how p63 regulates hair follicle development is not known yet. It has recently been shown that epidermal specific knockout of Dicer results in stunted development of hair follicles and hypo proliferation, reassuring a phenotypic match between the p63 and the Dicer null mice [20]. In connection with these findings, it has recently been shown that in the absence of Dicer or miR-203, ΔNp63 expression is not switched off in the suprabasal cells of the epidermis [8], indicating that Dicer-mediated miRNA processing is required for the down regulation of ΔNp63. Thus, it is plausible that p63 and Dicer could regulate each other to control the hair follicle and epidermal development.

Next, I have investigated whether p63 could function as a regulator of the Microprocessor component Drosha. p63/ΔNp63 has been shown to bind to Smad5 [21], suggesting a possibility that it could regulate the signaling cascade mediated by Smad5. Interestingly, it has recently been shown that Smad5/1 interacts with the Drosha/p68, and thereby promotes the processing of pri-miR-21 [22]. These findings suggest that ΔNp63, by binding to Smad5, it could promote the association between Drosha and pri-miRNAs. This will result in increased processing efficiency of growth promoting pre-miRNAs, including pri-miRNA-21. Further, Foxo3a has recently been shown to suppress the expression of miR-21. Another study showed that p53 suppresses the expression of Foxo3a through its transcriptional target HDM2, suggesting that p53/TA-p73 could induce the expression of miR-21. Interestingly, miR-21 has been shown to suppress the expression of TA-p63/p53 [5], indicating a feedback loop between ΔNp63/p53/TA-p63/p73 and miR-21[Figure 1]. Thus, ΔNp63, by increasing the expression of mature miRNA-21, it could suppress the expression of tumor suppressor genes, including TA-p63 and p53 [Figure 1].

Further, Dicer expression appears to be down regulated in metastatic prone tumors [23], indicating that it could function as a tumor/metastasis suppressor. Interestingly, p63/p73 heterozygous mice are prone to metastasis, indicating that the tumor suppressor TA-p63/p73 could function as a positive regulator of the suppressor of metastasis Dicer. These reports prompted me to analyze the Dicer promoter sequence for p63 responsive elements. Evidently, I have found two perfect p63/p53-REs half sites and eight nearly perfect p63/p53-REs in the Dicer promoter [Table 1], indicating that it could be a direct transcriptional target of p63/p53/p73. In support of this data, a study showed that Dicer is differentially expressed in response to TA-p63 expression, indicating that it could indeed be its transcriptional target [Figure 1]. Remarkably, I have also found p53/p63-REs in the promoters of a number of other miRNA-processing components [Table 1] [Table S1], suggesting that they could be direct transcriptional targets of p53/p73/p63.

**Table 1:** The promoters of the key components of the miRNA processing machinery contain p53/p63-REs.

miRNA processing components	Position in the promoter sequences (human)	p53/p63-REs present in the promoters of the microRNA-processing components
Drosha or DNASEN	−2695 to −2672−2025 to −2000−1996 to −1970−1832 to −1797−1868 to −1841−1282 to −1253−1000 to −975	(**ctacaggctc**)atc(**caccatgccc**)(**ccccttgaac**tcaaca**ggtcatgtgg**)(**ccccttgaac**)tcaaca(**ggtcatgtgg**)(**gatcatggct**)cactgc(**agccttgga**)(**ctcctgggtt**)(**gggcttgtca**gttgccc**agcctggaaa**gcagt)(**atccatgggc**)ctcctttgt(**tgacttggaa**)(**tagctgggta**)cggtg(**atgcgtgcct**)
DGCR8	−2845 to −2821−2797 to −2777−2590 to −2570−1346 to−1320−500 to −475−225 to −203−47 to −6	(**acgcatgtg**)catg(**catgcgtgttc**)(**gtgcgtgtgt**)(**ctgcatgtgt**)(**tatctggacc**)(**agtcatggct**)(**gggcttgtaa**)aactct(**ggtcttgtaa**)(**ggccttgaat**)ttccc(**ctacgaggtat**)(**tagcttggga**)agcttc(**cgccgagggt**)(**gagcatgagc**)gccaggg(**gctctggtgt**)ctgaa(**cagcgtgttt**)
Dicer*	−3127 to −3107−3362 to −3342−2071 to −2051−1999 to −1972−1961 to −1933−740 to −728−386 to −362−347 to −319−256 to −246−211 to −202	(**aggcatggtg**)(**atgcgtgctg**)(**aggcgtggtg**)(**gtgcacgcct**)gta(**gtcccagtta**)(**acacttggat**)t(**gcacttgagg**)(**acccgtggac**)tatctga(**aatcaggaga**)(**aaacttgccc**)aaggggta(**gcccaagagg**)(**aagcaatagc**)ct(**tagcatgttc**)(**tgcctgggat**)tcca(**acccgtgaag**)(**ctgcaggatg**)cctgcaag(**cttcgtgacc**)(**aaccatgtcc**)(**aaacttgctt**)
TARBP2	−3761 to −3734−3334 to −3353−3314 to −3294−2952 to −2932−2787 to −2763−2632 to −2614−2437 to −2403−2155 to −2135−998 to −970−598 to −565−33 to −3	(**tcacttggtc**)ttgttac(**gggcatgatt**)(**atgcatgagt**)(**ggctggcgt**)(**tcactggaag**)(**aagcatggca**)(**tagctggtgt**)(**ctccatgttg**)(**ggacaggaag**)(**gctcaggggg**)(**cacttgagt**)(**tggcttgaga**)cg(**actctgggtg**)(**ggccttggtt**)ac(**cagcaagtct**)ca(**actcttgttt**)(**gggcatgcag**)(**gaacaggtgc**)(**gatcttgcca**)gctctcct(**tcccttgatt**)(**actcatggaa**)ag(**gaacaagacc)accaaggtg**)**cgccatgaag**ct**tct(cgtg)(cgtg)(cttg)acc**

p53/p63/p73 may regulate the microRNA processing components through its responsive elements present in their promoters.

* Microarray data suggest that this gene is induced in response to TA-p63/ΔN-p63 expression (Promoter sequence of this gene serves as a positive control).

The RNA binding protein, P2P-R, has been shown to bind to Dicer/TARBP2/Ago2, and thereby enhances the processing of mature miRNAs [24], [25] [Figure 1]. Inhibition of P2P-R appears to inhibit the production of mature miRNAs, indicating that it could be part of the RNA-induced silencing complex. It also appears to enhance the siRNA-induced RNA interference. Bioinformatics analysis of P2P-R promoter suggests that it contains five nearly perfect p63/p53- REs[Table S1], indicating that it could be a transcriptional target of p63. Evidently, a study showed that P2P-R is differentially expressed [3.2 fold] in response to ΔN-p63 expression, indicating that it could indeed be its transcriptional target. Interestingly, P2P-R has been shown to promote the binding of MDM2 to the tumor suppressor p53, and thereby interferes with its ability to transactivate its target genes [26] (discussed in detail later). Therefore, these data suggest that ΔN-p63, by increasing the expression of P2P-R, it could increase the processing of Pre-miRNA into mature miRNAs (eg. Oncogenic miR-21) [Figure 1]. Taken together, p63, by increasing the expression of the miRNA processing components [Table 1], it could enhance the processing of miRNAs and siRNAs.

### p73 is a regulator of microRNA processing complex

The tumor suppressor p73 gene is located on chromosome 1p36.3, while Argonaut genes, Ago1, Ago3, and Ago4, are located on chromosome 1p34-35. Interestingly, 1p34-35 region is shown to be lost in Wilms tumors [27], suggesting that the Agos may function as tumor suppressor genes. Further, there seems to be a phenotypic similarity between Dicer and p73 null mice. Both p73 null and Dicer-hypomorphic mice are: a) hyper susceptible to infections; and b) infertile [28], [29]. In addition, lung specific Dicer knock out mouse model suggests that it is essential for lung morphogenesis [30], while TA-p73 specific knockout mice suggests that it is essential to inhibit the development of lung carcinoma.

**Figure 2:**
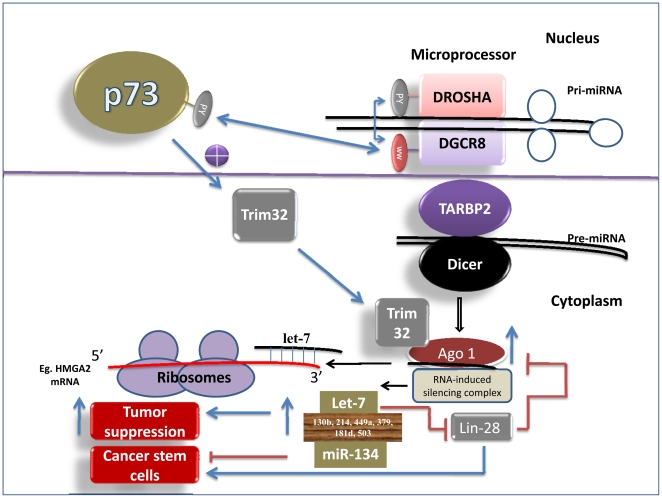
p73 is a regulator of microRNA-processing complex.

It has recently been shown that the ubiquitin ligase TRIM32 binds to Ago-1[31]. The C-terminal NHL domain of TRIM-32 forms complex with Ago1, and thereby promotes the efficiency of processing of a number of miRNAs [Figure 2], including let-7, miR-134, miR-130, miR-214, 449, 379, 181, and miR-503 [31]. To find out whether the tumor suppressor TA-p73 could increase Ago-1 expression through TRIM32, I have analyzed both human and mouse TRIM32 promoter sequences for putative p53/p63-REs. Remarkably, I have found five p63/p53-REs both in the human and mouse TRIM32 promoters, indicating that it could be a transcriptional target of TA-p73/p63; and p53 [32]. Evidently, a recent microarray study suggests that TRIM32 expression is increased in response to TA-p73 expression [33]. Together, these data suggest that the TA-p73, by increasing the expression of TRIM32, it could increase the Ago1/2 activity [Figure 2]. Interestingly, a domain present near the carboxyl terminus of p73 is homologous to enodonuclease III. Thus, it would be interesting to test whether this domain participates in Ago-1′s ability to enhance the miRNA processing. In addition, both Ago-1 and -2 promoters appear to contain p53/p63-REs [Table 1], suggesting that they could also be direct transcriptional targets of p53/TA-p73/p63. Thus, p73, by increasing the expression of Ago-1/2, it could increase the processing of miRNAs, such as let-7 (HMGA2; lin-28; EGFR; Kras; c-myc; Bcl-xL), miR-134 (Nanog; LRH1; Oct-4; Collagenase-3; Stromelysin), miR-130b (ERK2; Fosl1; TGFβR1; ERα; Tcf-4; Collagenase-3; Ago4; Dicer; p63), miR-214 (EZH2; CTNNB1), miR-449a (CDK6; SirT1; HDAC1; E2F-1), miR-503 (CCND1; Fosl1), miR-181d (ERK2; TGFβR1; Tcl-1; ERα; AID; Bcl-2) and miR-379 (lin-28) [Figure 2] [31], [32]. Increased expression of these miRNAs will activate tumor suppressor mechanisms and thereby suppress Epithelial to Mesenchymal transition (EMT), metastasis, and cancer stem cell (CSC) proliferation [Figure 2] [32].

Further, like p63, p73 contains the PY domain [34], suggesting that it could interact with the WW domain of DGCR8, and thereby influence its ability to process miRNAs [Figure 2].

It has recently been shown that let-7 suppresses the expression of Dicer, which, in turn, suppresses the expression of let-7, indicating a double negative feedback loop [35], [36]. This data suggests that TA-p73, by increasing the expression of the tumor suppressor let-7, it could indirectly regulate the expression of Dicer. It may even directly regulate the expression of dicer through its responsive elements present in the dicer promoter in a cell context dependent manner [Table 1]. In addition, both let-7 and lin-28 (an RNA binding protein) have been shown to inhibit each other's expression [37], [38]. This finding suggests that TA-p73/p63/p53, by suppressing the expression of lin-28 through let-7, it could regulate the miRNA-processing efficiency.

It has recently been shown that TA-p73 null mice are prone to lung cancer, indicating that it functions as a tumor suppressor gene [29], [39], [40]. Dicer gene is shown to be located on chromosome 14q32.13, while p73 gene is located on chromosome 1p36.3. Interestingly, these chromosomal loci are frequently lost in a number of cancers, including lung cancer. Evidently, Dicer expression appears to be lost both in advanced invasive lung cancer [41] and in non-small cell lung cancer [42]. In support of these findings, a study has recently found heterozygous germline mutations in the Dicer gene in a pediatric lung carcinoma [43], indicating that decreased expression of Dicer may serve as an initiating event for the development of lung carcinoma and metastasis. It has been shown that EMT promotes metastasis, self-renewal, and migration of cancer stem cells. Interestingly, Dicer expression appears to be lost in epithelial component, but retained in mesenchyme, suggesting a disruption of homeostatic mechanisms [43]. In support of this finding, a recent study suggests a lower expression of Dicer is associated with the mesenchymal phenotype [44]. Interestingly, this has matched in patients with the metastatic relapse of breast cancer [44]. These data suggest that Dicer could function as a metastasis suppressor gene. Intriguingly, p73/p63 heterozygous mice are more prone to sarcoma (mainly originates from mesenchymal cells) and metastasis when compared to p53 heterozygous mice, indicating that p73/p63 could function as a suppressor of sarcoma and metastasis. Evidently, TA-p73/p63 appears to increase E-cadherin expression (a negative regulator of EMT), by suppressing ZEB1/2 through its target miRs, such as miR-192, miR-215, miR-145, miR-203, miR-200b, miR-200c, miR-183, miR-92a/b, miR-132, and miR-30a-e [45]. Therefore, these data suggest that p73/p63, by increasing the expression of Dicer in epithelial cells, it could prevent the development of sarcoma and metastasis. Together, these data support the notion that TA-p73/p63 could function as a metastasis suppressor.

### p53 is a regulator of microRNA processing complex

It has been shown that Dicer is over expressed in prostate cancer and in the initial stages of adenocarinoma [1], [41], suggesting that it is deregulated in a tissue/stage specific manner in cancer. As discussed, Dicer appears to be required for the increased expression of self-renewal factors, indicating a pro-proliferative role for dicer [8]. However, Dicer also appears to play a tumor suppressor role. This is evident from the following studies: a) Ablation of Dicer in a k-ras induced mouse model of lung cancer enhances tumorigenesis. b) Mutation in TARBP2 protein destabilizes Dicer protein and promotes tumor growth. Conversely, reintroduction of a wild-type TARBP2 increases the Dicer stability, and thereby inhibits tumor growth [46]. c) Hepatocytes specific knockout of Dicer in mice promotes hepatocellular carcinoma after one year [47]. In addition, conditional knockout of Dicer both in liver and lung promotes apoptosis [30], [47], suggesting that increased expression of pro-apoptotic factors, such as tumor suppressor genes, may cause apoptosis. Together, the role of Dicer in the control of cell proliferation remains nebulous.

Further, targeted deletion of Dicer/DGCR8/HDM4 (the negative regulator of p53) in heart results in dilated cardiomyopathy [48], [49], [50]. This data suggests that constitutive expression of p53–in the absence of its negative regulator MDM4–in cardiomyocytes may promote apoptosis by suppressing the expression of Dicer. Perhaps this also explains why mice lacking Dicer and DGCR8 are developing dilated cardiomyopathy. Of interest, a mutation in DGCR8 gene is characterized by congenital heart defects [9].

These reports prompted me to surmise that Dicer could be a transcriptional target of p53/p63/p73. As discussed, Dicer promoter contains several p53-REs, indicating that it could be a transcriptional target of p53/p63/p73. However, analysis of its p53-REs sequence [Table 1] suggests that—“CGTG”: p63 specific; CATG: p53 specific— it could be a preferential transcriptional target of p63/p73 rather than p53.

Further, p53 appears to regulate the expression of Dicer through its transcriptional target miRs, such as miR-192, 215, miR29a/b/c, miR-148, miR-15/16a, miR-206, and miR-103 [Table 2], suggesting that p53/p63/p73 could regulate the expression of dicer both at the transcriptional and the post-transcriptional level. Interestingly, these p53-miRs have been shown to promote growth arrest and apoptosis. Considering the tumor suppressor p53 is a negative regulator of stem cell proliferation and reprogramming factors (eg. Nanog, Oct-4, Klf4, Sox-2, lin-28, and c-Myc, Boominathan, published elsewhere) [32], it is unsurprising that it could function as a negative regulator of Dicer.

**Table 2:** p53/p63/p73-microRNAs that target essential components of the miRNA processing machinery.

miRNA processing components	Target miRNAs	Target scan's Context percentile/Mami score(in decimals)/miTG score (*in italics*)	Transcriptional targets of p53/p63/p73/target miRNA- processing components/comments
DROSHA or DNASEN	miR-27a/bmiR-128	8340	p53p53
DGCR8	miR-9miR-27amiR-31	67*8.25* *67*	p53p53p73/p63/p53
Dicer	miR-103miR-107miR-16*miR-15*miR-206miR-29a/b/cmiR-192*miR-215*let-7 a to gmiR-124miR-200amiR-130a/bmiR-203miR-31	95*33.45*524289808686*31.95 to 8.6* *14.2* *7.8* *82/75* *72*73	p53p53p53p53/p73p53p53; increases p53 expressionp53p53p73/p63/p53p53p53p73p53/p73/p63p73/p63/p53
TARBP2	Let-7a to gmiR-15a/b*miR-16*miR-195miR-103miR-107miR-183*miR-34b*	66–728689890.094320.094320.094320.09432	p73/p63/p53p53/p73/p63p53/p73/p63p53/p73/p63p53p53p53/p73p53/p73
Exportin 5	miR-34a*miR-143miR-124miR-29a/b/c	9262*12.72* *10.25 to 10.13*	p53/p73p53p53p53

p53/p63/p73 proteins increase the expression of miRNAs to regulate the components of the miRNA-processing complex [56, 69, 70, Boominathan, unpublished].

* Experimentally verified transcriptional targets of p53.

It has recently been shown that Dicer is required to maintain hypermethylation in cancer cells[51], indicating that aberrant expression of Dicer could deregulate the expression of tumor suppressor genes in cancer cells. Evidently, knock down of Dicer results in up regulation of the tumor suppressors p53 and p19ARF, which in turn promote senescence [52]. Deletion of Dicer has also been shown to result in up regulation of the tumor suppressor p130, which in turn represses the expression of DNMT3a and DNMT3b [53]. This appears to occur through down regulation of miR-290, which functions as a suppressor of the expression of p130. Together, these data suggest the following: a. p53 and Dicer may share a reciprocal relationship. b. p53 may fine-tune Dicer expression in a cell context dependent manner. c. Increased expression of Dicer may either promote or inhibit tumorigenesis and metastasis in a cell context dependent manner. d. p53′s ability to regulate Dicer expression could be part of its intrinsic tumor suppressor mechanism.

Under physiological conditions, p53 will be in its inactive form, thus its transcriptional target miRs will be poorly expressed. This will result in stabilization of Dicer, which in turn, will promote the processing of growth promoting miRNAs. While in response to stress, p53 will be active, thus its transcriptional target miRs will repress Dicer function. This notion is supported by data showing that down regulation of Dicer expression elicits DNA damage response [54]. This results in activation of the tumor suppressors p53 and ARF. Of importance, it has recently been shown that the ability of some of the miRNAs to cause translational repression of their targets is compromised in response to stress [55]. However, this does not explain how stress related miRNAs, such as miR-34, miR-192, and miR215, are produced in a p53-dependent manner in response to DNA damage. If these miRNAs are processed independent of Dicer function or regulated in a context dependent manner, then one can explain this conundrum. Therefore, it would be fascinating to test how TA/ΔN-p63/p73/p53 and Dicer regulate each other both in response to DNA damage and under physiological conditions.

MiRNAs have been shown to inhibit the expression of the tumor suppressor p53 (miR-125a/b) and p63 (miR-92, miR-21, 302 and miR-203) [Table 3], indicating that Dicer function may be required to generate mature miRNAs. Besides, miR-150 and miR-22 are predicted to inhibit the expression of p53 [Table 3]. However, it is important to keep it in mind that miR-150 is expressed only in mature lymphocytes. Interestingly, p53 has been shown to suppress the expression of miR-125b and miR-22 [56], indicating that it could repress its negative regulators to increase its expression.

**Table 3:** p53, p63, and p73 are negatively regulated by miRNAs.

p53 related proteins	Targeting miRNA (verified)	Putative miRNAs that target p53 related proteins (human and mouse)
p53	miR-125a/b	miR-150; let7-i; let-7f; miR-98; let-7c,b, a, g, d; miR-22; miR-612
p63	mirR-302 and miR-21	miR-92; miR-101; miR-519C-3p; miR-519a; miR-519b-3p; miR-301a/b; miR-454; miR-130a/b; miR-367; miR-363; miR-372; miR-373; miR-377; miR-543; miR-590-3p; miR-340; miR-137; miR-543; miR-590-3p; miR-32; miR-495; miR-181c; miR-181a; miR19a/b
ΔN-p63	miR-203 and miR-92	-
p73	-	miR-125a/b; miR-205; miR-485-5p; miR-193a/193a-5p; miR-612

miRNAs targeting p53, p63, and p73 were analyzed using the Target scan, Diana and Mami softwares.

Let-7 miRNA is highly conserved from lower organisms to humans. A number of studies suggest that it function as a tumor suppressor gene. Estradiol, an inducer of p53 expression, has been shown to increase the expression of eight members of the let-7 family. Evidently, let-7a (2.1 fold), let-7c (2.7 fold), and let-7e (2.1 fold) are induced in response to p53 expression [56]. Remarkably, the let-7 family promoters contain p53-REs [40], indicating that they could be indeed be direct transcriptional targets of p53/p73/p63. Surprisingly, let-7c (Context score percentile: 87) and let-7a (Context score percentile: 87) are predicted to target the expression of p53 (Target scan) [Table 3]. This data suggests a) a feedback loop between p53 and let-7; and b) the ability of let-7c and -7a to inhibit p53 expression may be relieved in response to DNA damage [57]. Evidently, let-7 has recently been shown to increase the expression of its target mRNAs under stressful conditions [57]. Thus, this data suggests that let-7 may increase the translation of p53 mRNA under genotoxic conditions.

**Figure 3:**
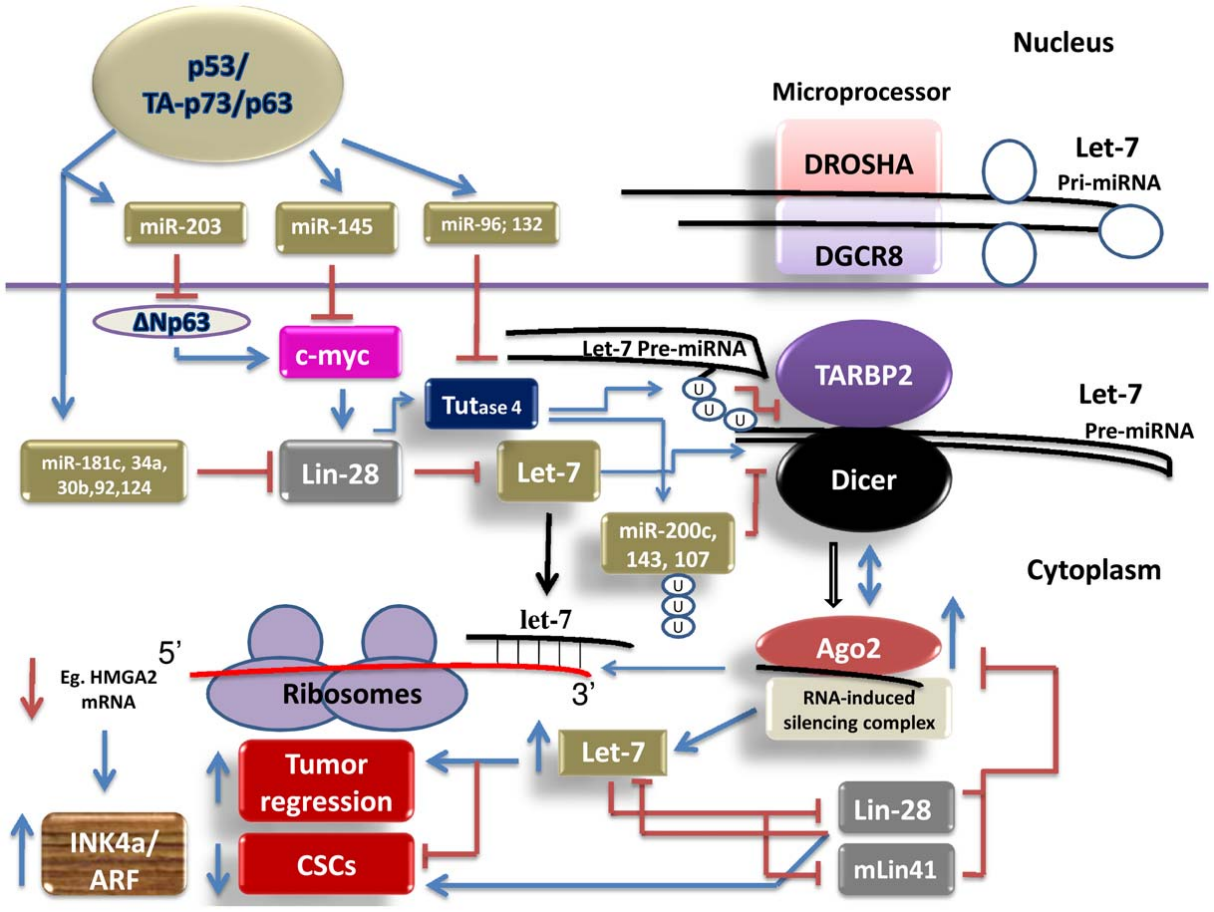
p53, TA-p73/p63, and ΔNp63 regulate the processing of the tumor suppressor miRNAs, let-7, miR-200, miR-143, and miR-107.

The stem cell renewal factor, lin-28, has been shown to suppress the processing of pre-let-7 at the Microprocessor step [37]. It interacts with let-7 precursor loop, and thereby inhibits its processing in embryonic stem cells [58]. It has also been shown to mediate uridylation of let-7 at its 3′ end in the cytoplasm [59]. The uridylated let-7 fails to be processed by the Dicer complex and therefore undergoes degradation in the cytoplasm. However, the enzyme that uridylates let-7 precursor remains obscure. Remarkably, Heo et al., [60] have recently showed that lin-28 recognizes a sequence motif—GGAG—present in let-7, and recruits TUTase-4 [TUT4, uridyl transferase]. The TUTase4 uridylates let-7 precursor, and thereby inhibits its processing [Figure 3]. Further, Let-7 has recently been shown to reduce the expression of stem cell reprogramming factors, such as Klf-4, Oct-4, Sox-2, and c-Myc [59], [61]. Interestingly, knockdown of lin-28 and TUTase4 results in down regulation of stem cell markers, indicating that both lin-28 and TURase4 are required for the stem cell maintenance [60]. As discussed, p53/TA-p73/p63, by increasing the expression of let-7, it could suppress the expression of the stem cell reprogramming factor lin-28 [37], [38], [40]. Together, p53/TA-p73, by increasing the processing of the tumor suppressor let-7, it could inhibit the stem cell proliferation/renewal/maintenance.

Further, p53-miRs, such as miR-96 and miR-132 [56], are predicted to target the expression of TUTase4 (Target scan) [Table 2]. This data suggests that p53/TA-p73/p63, by suppressing the TUTase4 expression through its target miRs, it could increase the processing of the tumor suppressor let-7. This data further strengthens the notion that p53/TA-p73/p63, by increasing let-7 expression, it could reduce stem cell proliferation/renewal/maintenance [Figure 3].

Lin-28 may also mediate uridylation of other miRNAs, such as miR-143, 200c, and 107, as these miRNAs share the sequence motif GGAG. By uridylating these miRNAs, lin-28 could inhibit their processing [58], [56] [Figure 3]. Remarkably, these miRNAs have been predicted to function as transcriptional targets of p53/TA-p73/p63 [45, 56, 69, Boominathan, unpublished]. A) miR-143 has been shown to inhibit the expression of DNMT-3a [62]. It also appears target the expression of k-ras, AID, and ERK2. Inhibition of DNMT3a may result in reactivation of tumor suppressor genes in cancer cells, while inhibition of ERK2 may result in down regulation of the EMT activator and the metastasis promoter ZEB1/Fosl1 expression. B) The tumor suppressor miRNA, miR-200c has also been shown to inhibit the expression of ZEB1 [45]. Interestingly, ectopic expression of miR-200c has been shown to inhibit lung carcinoma cell lines' ability to undergo EMT, invade, and metastasize. Considering ZEB1 is a suppressor of E-cadherin, TA-p73 and p21 expression, increased expression of miR-200c (an inducer of epithelial phenotype) will result in increased expression of the tumor suppressor E-cadherin/TA-p73/p21 and in turn, this will result in inhibition of proliferation, EMT, invasion, metastasis and CSCs proliferation [45], [63], [64], [65], [66], [67]. C) miR-107 has recently been shown to promote cell cycle arrest [68]. It also suppresses HIF-1β and VEGF expression, and thereby inhibits tumor angiogenesis. Together, these data suggest that p53/TA-p73/p63, by negatively regulating the expression of lin-28/TUT4ase through its target miRs, it could increase the processing of the tumor suppressor-miRNAs, let-7, miR-200c, miR-143, and miR-107 [Figure 3]. Increased expression of these tumor suppressor miRNAs will result in inhibition of tumorigenesis, EMT, invasion, and metastasis.

Further, p53-miRs, such as miR-181c, 30b and 92 [Table S2], are predicted to target lin-28B [56], [69], [70], indicating that p53, by suppressing the expression of lin-28B, it could increase the processing of the tumor suppressor miRNA, let-7. Interestingly, increased expression of let-7 has been shown to increase the expression of Ago-2 [71]. This data suggests that p53/TA-p73/p63 could increase the expression of Ago-2 through its target gene let-7, and thereby it could increase the processing efficiency of miRNAs [Figure 3].

miRNA-145, a transcriptional target of p53/TA-p73/p63, has recently been shown to suppress c-Myc (a transcriptional activator of lin-28), metastasis promoting factors (Fascin, mucin1), and stem cell reprogramming factors (Oct-4, Klf-4, Sox-2) [72], [73]. This data suggests that p53/TA-p73/p63, by negatively regulating c-Myc/lin-28 through its transcriptional target miR-145, it could increase the expression of let-7 [73]. HMGA2 has been shown to suppress the expression of INK4a/ARF expression [74]. Interestingly, let-7 has recently been shown to suppress the expression of HMGA2, suggesting that p53/TA-p73/p63, by increasing the expression of let-7, it could increase the expression of INK4a/ARF (TAp73/p63-let-7-HMGA2-INK4a/ARF) [Figure 3]. This notion is in concordance with the tumor suppressor—E2F-1-TA-p73/p63-JunB-INK4a/ARF– pathway that I have proposed previously [39], [40]. HMGA2 has also been shown to function as an activator of Snail1 and Slug (Snail2) expression (inhibitors of E-cadherin/miR-200 expression), suggesting that p53/TA-p73/p63, by suppressing the expression of both Snail and Slug, it could induce the expression of E–cadherin and miR-200. Together, p53/TA-p73/p63, by increasing the expression of miR-145, let-7, and miR-200, it could decrease the expression of metastasis promoting factors, and stem cell factors.

Further, a microarray study suggests that ΔN-p63 expression increases the expression of c-Myc [75]. This data suggests that ΔN-p63, by increasing the expression of c-Myc, it could increase the expression of lin-28. Increased expression of lin-28 will suppress the processing of let-7 pre-miRNA. Interestingly, ΔN-p63 expression has shown to be suppressed by miR-203, a transcriptional target of p53/TA-p73/p63 [76] [Figure 3]. This data suggests a double negative feedback regulation between ΔN-p63 and p53/TA-p73/p63 [76]. Further, mLin41/TRIM71, an E3 ubiquitin ligase, has recently been shown to mediate Ago-2 ubiquitylation [77], and thereby reduces the silencing efficiency of miRNAs. Interestingly, let-7, by suppressing mLin41 expression, it could enhance the Ago-2-dependent silencing efficiency of miRNAs. Together, these data suggest that both p53/TA-p63/p73 and ΔN-p63 could regulate the processing of tumor suppressor let-7 through their transcriptional targets in a cell context dependent manner [Figure 3].

**Figure 4:**
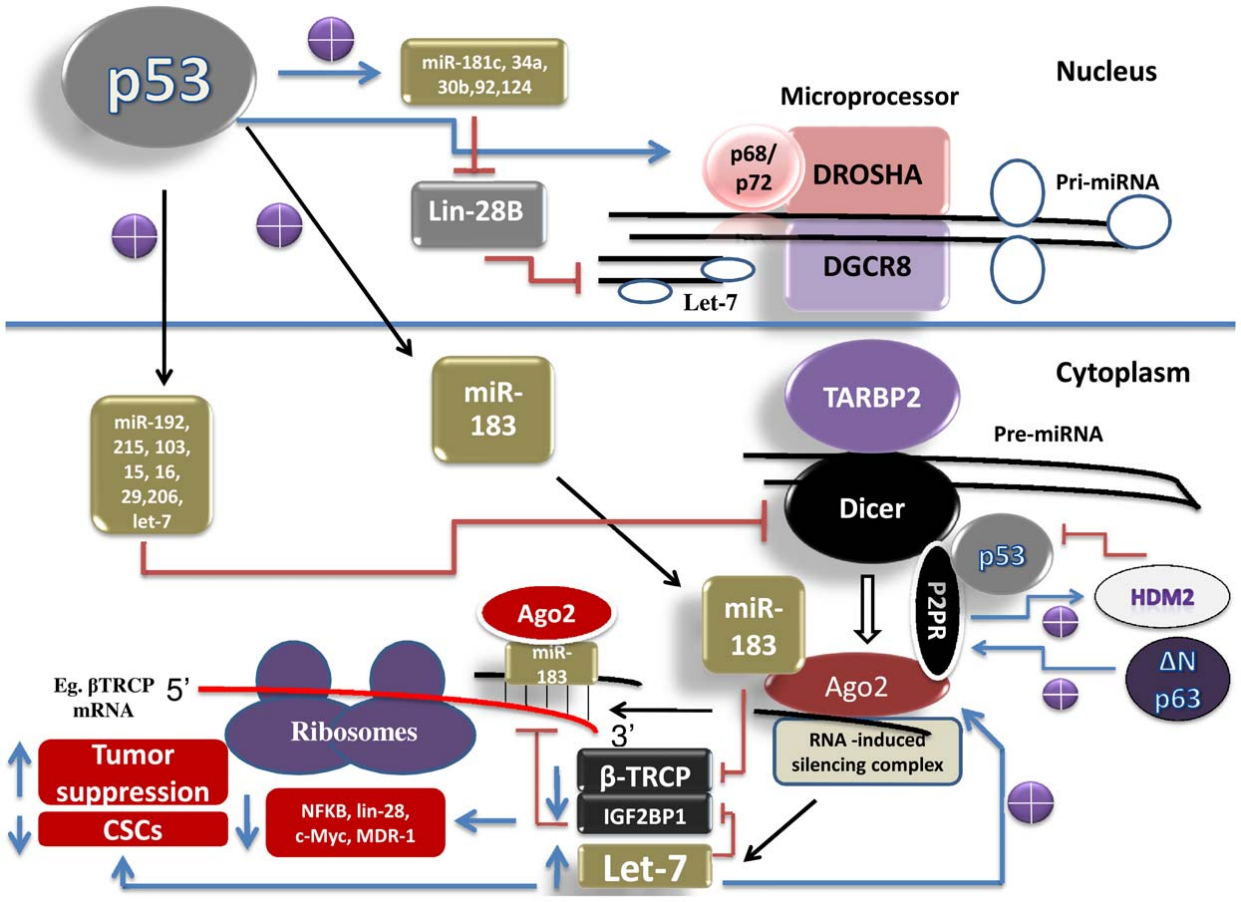
p53 is a regulator of microRNA-processing complex.

miR-183 has recently shown to be induced in response to p53 expression [56]. Evidently, bioinformatics analysis of its promoter revealed a number of p53-REs, suggesting that it could be a transcriptional target of p53 [Boominathan, unpublished]. It has recently been shown that miR-183 binds to the coding region of β-transducin repeat-containing protein 1 [βTrCP1] mRNA, a substrate recognition protein of the SCF E3 ubiquitin ligase complex, and thereby destabilizes it by recruiting the Ago-2 containing RNA-induced silencing complex [78], [56] [Figure 4]. Interestingly, both Ago-2 and miR-183 bind to the over lapping region of βTrCP1. Furthermore, IGF2BP1 has been shown to bind to the coding region of βTrCP1, c-myc, and MDR-1 mRNAs, and thereby protects them from degradation [78]. Remarkably, let-7 has recently been shown to suppress the expression of IGF2BP1, suggesting that it could destabilize βTrCP1, c-myc, and MDR-1 mRNAs. miR-183 also appears target the expression of ZEB1 [45], TCF-4, and NFKB1, an activator of lin-28, IL-6 and Snail1 (inducers of EMT & metastasis) expression(Target scan). Together, these data suggest that p53, by inducing miR-183 and let-7, it could promote the destabilization/degradation of oncogenic mRNAs (βTrCP1, c-myc, MDR-1, IGF2BP1, ZEB1, TCF-4, NFKB1) and thereby suppress the expression of metastatic factors [79] [Figure 4].

p68 is an ATP-dependent RNA helicase that helps in unwinding stem loop containing pri-miRNAs. It appears to be part of the Drosha complex. It has been shown to unwind the stem loop structures of the tumor suppressor miRNA, let-7 [80]. It has also been shown to function as a co-activator of p53 in response to DNA damage [81]. Interestingly, p68 promoter contains p53-REs [Table S1], indicating that p53 may increase its expression to increase the processing efficiency of the Drosha complex [Figure 4].

As discussed before, P2P-R has been shown to interact with integral components of the RNA-induced silencing complex, such as Dicer, TARBP2 and hAgo-2. It also interacts with p53 and HDM2, indicating that p53 could either be part of the RNA-induced silencing complex or it could regulate the formation of the RNA-induced silencing complex. Interestingly, ΔN-p63, which has been shown to antagonize p53/TA-p63/p73 functions, appears to increase the expression of P2P-R [Figure 4]. Increased expression of P2P-R has been shown to augment HDM2′s ability to ubiquitinate p53, and thereby promotes it for degradation [26]. This data suggests that ΔN-p63, by increasing the expression of P2P-R, it could decrease the p53 protein levels. ΔN-p63-mediated down regulation of p53 and its target miRNAs may result in increased expression of Dicer [Figure 4]. It is possible that this regulatory mechanism is functional only under proliferation promoting conditions. Remarkably, p53-miRs, such as miR-15, 16, and 29a/b/c, are predicted to inhibit the expression of P2P-R [Table S2], suggesting that there could be a feedback loop between p53/ΔN-p63 and P2P-R [Figure 4]. Interestingly, miR-15/16/29 promoter contains p53/p63-REs, suggesting it could be a direct transcriptional target of p53/TA-p73/p63 (Boominathan, unpublished).

It has been shown that NF90/ILF-3 and NF45/ILF-2 function as RNA/DNA binding proteins. Depletion of these proteins appears to cause growth inhibition [82]. Interestingly, it has recently been shown that both NF90 and NF45 function as negative regulators of the miRNA processing [83]. They do not appear to bind to any of the known miRNA processing components, but they interact with pri-miRNAs of let-7 and miR-21. By interacting with let-7/miR-21 pri-miRNAs, they inhibit the processing of let-7/miR-21 pri-miRNAs into pre- miRNAs. Interestingly, both ILF-3 and ILF-2 promoters contain p53-REs [Table 1], suggesting that they could be direct transcriptional targets of p53 or p53 related proteins. Further, ILF-3 and ILF-2 are targeted by miR-181b/c and miR-25, respectively [Table S2]. Together, these data suggest that both p53 and p53-miRs, by controlling the expression of both ILF-3 and ILF-2, they could regulate the processing of let-7/miR-21 pri-miRNAs.

Estrogen receptor-α (ER-α) has been shown to attenuate the Drosha-mediated cleavage of pre-miR-145/16/143 [84]. Interestingly, p53-miR-145 has recently been shown to inhibit the expression of ER-α [85], suggesting that p53/p73/p63 could function as a positive regulator of the processing of the tumor suppressor miRNA-145/16/143. As discussed, p73 may inhibit the expression of ER-α through its ability to increase the processing of miR-130b/181d. Further, hnRNP A1, an RNA binding protein, has been shown to interact with conserved loops of pre-miR-18a and thereby promotes its cleavage mediated by the Drosha complex [86]. Interestingly, p53 appears to inhibit the expression of hnRNP A1 through its target miRNA-15/16, suggesting that p53/p73/p63 could function as a negative regulator of the processing of the oncogenic miRNA-18a. Together, these data suggest that p53, p73, and p63, by controlling the expression of auxiliary factors necessary for the processing of specific miRNAs, they could function either as a positive or as a negative regulator of the miRNA processing in a cell context dependent manner.

### p53/p63/p73-miRNAs' ability to target the components of the miRNA processing complex are conserved

Among the p53-miRs that target the components of the miRNA processing complexes, miR-15/16/195, miR-103, miR-107, let-7, miR-124, miR-181, miR-148a/b, miR-30a/c, miR-27, miR-17, and miR-20 appear to target more than five components of the miRNA-processing pathway [Table 4, Table S3], suggesting the conserved nature of p53-miRs. In response to p53 activation, miR-17, miR-20, miR-27a, miR-15a, miR-15b, and miR-16 are induced 4, 7.7, 5.2, 5.2, 8.2, and 2.9 folds respectively, compared to controls [56].

**Table 4:** p53-microRNAs that target multiple components of the miRNA processing machinery.

Components of miRNA- processing complex	p53-miRs that commonly target components of the miRNA processing complex	Target scan/Mami(in decimals)/miTG(*in italics*) score (human)
TARBP2DicerLin-28Lin-28BAgo 3Ago 4mLIN41	let-7	66–72*31.95*939844–4960–6597
DicerRCKExportin 5Ago1TNRC6BLin-28b	miR-124	*14.2*96*12.72* *14.18* *32.36* *15.63*
KHSRPILF3RANLin-28Lin-28BmLIN-41	miR-181	-810.25860927595

As discussed, miRNAs are processed by the RNAse III containing complexes, such as Drosha-DGCR8 (in the nucleus); and Dicer-TARBP2 (in the cytoplasm). Interestingly, both Drosha and DGCR8 appear to be targeted by p53-miR, miR-27, while both Dicer and TARBP2 appear to be targeted by p53-miRs, such as let-7, miR-103/107, and miR-15/16/195, suggesting a co-ordinated regulation of miRNA processing mediated by the p53-miRs. The data presented in Table 4/Table S3 suggests the conserved nature of p53/p73/p63-miRs in targeting a number of components of the miRNA processing complex.

**Figure 5:**
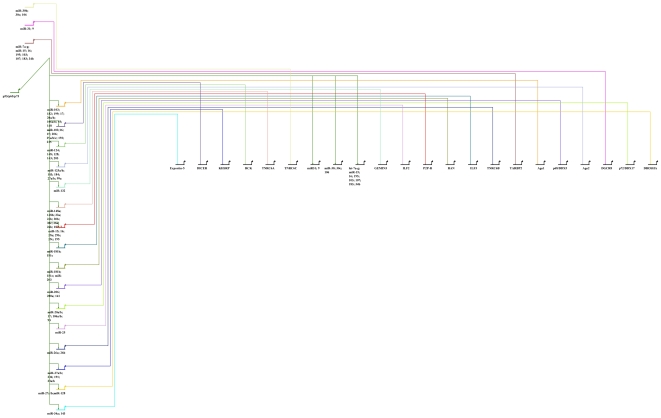
This working model shows how p53, p63, p73, and their target miRNAs regulate the microRNA-processing components. It was drawn using biotapestry software [87].

The miRNA processing components' function appears to be profoundly influenced by the p53, p63, and p73. Table 1 [Table S1] presents data that suggests miRNA processing components could be direct transcriptional targets of p53/p63/p73. Table 2 [Table S2] shows that the p53/p63/p73-dependent miRNAs could regulate the expression of the miRNA processing components. These data strongly suggest that p53, p63, and p73 may co-operate with each other in regulating the miRNA processing components. In addition, there could be positive and negative feedback loops between p53/TA-p63/p73/ΔN-p63/p73 [Table 3 and Figure 3] and the miRNA processing components [Figure 5] (production will need this reference to link the reader to the figure) [84]. Further, the data discussed in this article suggest that TA-p73/p63 is no longer a phantom of a tumor suppressor, but it is indeed function as a tumor suppressor.

Considering that a number of miRNAs function as tumor suppressor genes [32], [40], [45], [73], [76], it is of great importance to understand how the guardians of the genome, p53, p63, and p73 regulate the miRNA processing machinery. Given that a number of components–DGCR8, XPO5, TNRC6B, Ago1/2, TARBP2, HSPCA, SERBP1A, and Dicer–of the miRNA-processing complex are down regulated in cancer [1], it is unsurprising that they could be regulatory targets of the guardians of the genome. In conclusion, the data discussed in this article not only provide insights into how p53, p63, and p73 function as regulators of the miRNA processing machinery, but also how they inhibit tumorigenesis, EMT, metastasis and CSC proliferation.

## Materials and Methods

### Identification of p53/p63-REs present in the promoters of components of the miRNA complex

miRNA processing components' promoter sequences were downloaded from http://genome.ucsc.edu/. -5000bps upstream to transcription starting sites was analyzed for p53/p63REs using TRANSFAC. p53/p63-REs' [RRRCWWGYYY] core sequence is underlined.

### Curated p53-dependent or p53-inducing miRNA data

A list of p53-dependent or p53-inducing miRNAs (data not shown) was prepared from the published microarray data [56, 69, 70, Boominathan, unpublished].

### Identification of miRNAs using Target scan, Diana, and Mami bioinformatics tools

miRNAs that target miRNA processing components were analyzed using Target scan [http://www.targetscan.org/], Diana [http://diana.cslab.ece.ntua.gr/microT/] and Mami [http://mami.med.harvard.edu/] softwares.

**a. Target scan:** This site is used for the prediction of miRNA target genes. Importantly, it indicates whether the identified miRNA is conserved across several genomes. It also indicates the number of times the seed sequence of an miR matches with the 3′UTR sequence of mRNAs. Accuracy of the predicated target can be determined from the context percentile score.

**b. Diana:** This site also provides a list of predicted miRNAs for the chosen mRNA. This program appears to provide a highly sensitive score compared to other programs.

**c. Mami:** This site can be used to find out whether the identified targeting miRNA is predicted by more than one program. This site provides a comparison of five different programs, such as, Targetscan, miRanda, microT, miRtarget, and picTar. Mami score is presented in decimals (miTG).

Numerous miRNAs appear to target the components of the miRNA processing complex. However, only p53-dependent miRNAs were selected for further analysis (refer to Table 2 [S2] & 4 [S3]).

### Comparative/computational miRNA analysis

A p53-miR is considered conserved if it targets more than one component of the miRNA- processing complex. Keeping this as a criteria for selection, a list of p53-miRs that targets more than one component of the miRNA processing complex was prepared (refer to Table 4/S3).

## Supporting Information

Table S1 The miRNA biosynthesis pathway component's promoters contain p53/p63-REs.
(0.05 MB DOC)

Table S2 p53/p63/p63-miRs that target components of the miRNA biosynthesis pathway.
(0.05 MB DOC)

Table S3 p53-miRs that target more than one component of the miRNA processing machinery.
(0.05 MB DOC)
