# Supplementary material for: The Tumor Suppressors p53, p63, and p73 Are Regulators of MicroRNA Processing Complex
Source: PLoS One. 2010 May 12;5(5):e10615. doi: 10.1371/journal.pone.0010615 (PMC2868896; doi:10.1371/journal.pone.0010615)
Supplement: Table S1 — The miRNA biosynthesis pathway component's promoters contain p53/p63-REs. (0.05 MB DOC) [file pone.0010615.s001.doc]

Supplemental Table 1

| TARBP2 | -998 to -970  -598 to -565  -33 to -3 | **(gatcttgcca)**gctctcct(**tcccttgatt)**  **(actcatggaa)**1ag(**gaacaagacc)accaaggtg)**  **cgccatgaag**ct**tct(cgtg)(cgtg)(cttg)acc** |
| --- | --- | --- |
| p68 or DDX5 | -339 to-315  -104 to-161  -37 to -48 | **(cgccatgtcg)**ggtt(**attcgagtga)**  **(cggcttggac)**ttggcctttccgggc(**tatcttggga)**cttcctttcccga(**aggcttgcgc)**  **(aagcttgctt)** |
| p72 or DDX17 | -1130 to -1106  -566 to -594  -107 to -87 | **(gatcgtgcca)**ctcc(**agcctgggtg)**  **(gttcgagacc)**agcctgac(**caacatggag)**  **(gagcttggga)t(tttctggcac)** |
| EIF2C2/  Ago-2 | -577 to -555  -638 to -660  -1042 to -1005  -1296 to -1266  -2663 to -2743  -2400 to -2355  -1949 to -1918  -1701 to -1660  -1295 to -1265  -1042 to -1005 | **(agccaggctg)**gg(**ggccgagggg)**  **(gcgcgagcgg)**cc(**ggccgcgttc)**  **(cttcagggct)**gtc(**ctccgagact**g)caga(**gaactagtga)**  **(gtccaagcgg)**tgatgaagaa(**tgccatgcac)**  **(gaacatgttt)**taggaacaaatgatttattccattgcctgcatttt(**agtcttgaag)**aaacaaagtgcccccaa(**agccgagggc)**  **(ggacgtgggt)**ctgggggacagagcagcaagggctgg(**agacatgtca)**  **(tatcttgcct)**tctggaacgaa(**atccttgcaa)**  **(ttgcatggtt)**cataaataggaaagtggtaac(**attcatgatt)**  **(gtccaagcgg)**tgatgaagaa(**tgccatgcac)**  **(cttcagggct)**gtc(**ctccgagact)**gcaga(**gaactagtga)** |
| EIF2C1/  Ago-1 | -47 to -65  -538 to -528 | **(ttgcgtgat)(ggcgtgggt)**  **(aaacgtgtgt)** |
| EXPO5 | -296 to -277  -769 to -743  -1050 to -1027  -1553 to -1527  -1740 to -1717  - 2201 to -2167  -2292 to -2272  -3597 to -3576  -4210 to -4189 | (**agacgagagc)ggcgcgccc)**  **(aaacaagttt)**agtgcc(**caccaggtaa)**  **(caccatgttg)gtg(aggctggtct)**  **(ttacaagtta)aaacta(taacatgcca)**  **(atccatgatt)**taa(**atacgagaaa)**  (**aggcgtgagc)(catcgcgctc)ggcc(tagcatgggg)**  (**agactgggtt)t(caccatgttg)**  **(acacatggca)(aggcatgccc)**  (**atgcttgaag)g(cagcatgctc)** |
| KHSRP | -3360 to -3337  -3030 to -3012  -2120 to -2098  -979 to -958 | **(gtgctgggat)**tac(**aggcatgagc)**  **aggcttga(gggcatgttt)**  **(aggcatggta)**gtat(**ggacctgtgg)**  **(actcgtgagt)g**(**gggctggttc)** |
| NF90/ILF3 | -3594 to -3573  -2722 to – 2702  -1290 to -1263  -485 to -457 | (**aaacatgatt)**g(**gggcattagggc)**  **(gaacgagaag**)(**aggcgtgggc)**  **(tatcgtgatt)**tgccttt(**gaacttggtc)**  (**tggcaggtgc**c)(**ctccttggaa)actggttt** |
| NF45/ILF2 | -2247 to -2228  -1896 to -1873  -1851 to -1831  -580 to -560 | (**aggcatggtg)(gtgcatgcct)**  (**caacatggtg)**aaa(**ccccatgtct)**  (**gggcagggcc)(gggcatggtg)**  **(ggacatggtg)(gcgcatgcct)** |
| TNRC6A | -59 to -85  -191 to -230  -1288 to -1311  -1359 to -1395  -1553 to -1585  -2404 to -2424  -2532 to -2570 | (**catcttgggg)**gccagt(**ggccgtggcg)**  (**ctccagggag)**gtc(**cggctgggtg)(cagcgtgaca)**  (**agcctgggag)**aca**(gagcgagact)**  (**ctacttggga)**ggcta(**aggcaggaga)**a(**ttgcttgaac)**  (**aagcatgttg)**taaagccagttg(**aagctgggcg)**  (**aggcatgagc)(caccgcgcct)**  **(tagctgggat)**tac(**aggcatgcac)**cacta(**tgcctggcta)** |
| TNRC6B | -2341 to -2362  -2693 to-2711  -2765 to -2789  -3109 to -3132  -3287 to-3308  -3434 to -3458  -4212 to -4248 | **(aagcatgtcc)**atcacaaatta(**aaacaaggtg)**  (**gggcatggtggcatgtgt)**  (**tcacgaggtc)**agga(**gttcgagacc)**  (**agcctgggcg)**aca(**aagcgagact)**  **(tcacgaggtg)**a**(tcacgaggtg)**  **(caccatgcct)ggcc(aatcatgttt)**  (**gtgctgggat)**tac(**aggcatgagt)caa(caccatggcc)** |
| TNRC6C | -288 to -310  -332 to -352  -815 to -838  -856 to -879  -1647 to -1668  -4674 to – 4693 | (**ggacaagtca)**cc(**cacctggggt)**  **(tgccaagagt)(ggccatgctt)**  **(cttctggaa)cta(cagcaagtga)**  (**agcctggtgg)**tga(**acacatgaac)**  **(ggacatgaag)**g(**aaccgtggcg)**  (**caccatgttggccaggctg)** |
| RBQ1/  P2P-R* | -639 to -659  -877 to -897  -968 to -989  -1021 to- 1045  -2021 to-2051  -3062 to -3082 | **(taacgtgttt)(tgctgggat)**  (**aggcgtgagc)(caccgcgccc)**  **(agacagggtt)t(caccatgttg)**  (**tagctgggat)tacag(gcgcatgcca)**  (**gagcatgttt)(aacttggaa)(tggcaggcta)**  (**caacatgact)(caccaagact)** |

* Microarray expression data suggest that this gene is induced in response to TA-p63/ΔN-p63 expression.
