# Supplementary material for: The Tumor Suppressors p53, p63, and p73 Are Regulators of MicroRNA Processing Complex
Source: PLoS One. 2010 May 12;5(5):e10615. doi: 10.1371/journal.pone.0010615 (PMC2868896; doi:10.1371/journal.pone.0010615)
Supplement: Table S2 — p53/p63/p63-miRs that target components of the miRNA biosynthesis pathway. (0.05 MB DOC) [file pone.0010615.s002.doc]

**Supplemental Table 2**

| p68/DDX5 | miR-206  miR-200a  miR-141  miR-106a  20b  17 | 88  59  58  *12.61*  *8.24*  *8.14* | p53  p53  p53  p53  p53  p53 |
| --- | --- | --- | --- |
| p72/DDX17 | miR-20a/b  miR-17  miR-106a/b  miR-93  miR-9 | 81-83  77  77-78  76  *15.59* | Negatively regulated by p53 in response to DNA damage  Induces p73 expression  p53  p53 |
| EIF2C1/Ago1 | miR-103/107  miR-182  miR-17  miR-20a/b  miR-106a/b  miR-93  miR-148  miR-30c*  miR-29a/b/c  miR-206  miR-124 | 89  81  65  65  65  67  94  *21.46*  *15.79*  *14.6*  *14.18* | p53  p53  Negatively regulated by p53 in response to DNA damage(5-FU)  Positively regulates p73 expression  p53  p53  p53  p53  p53  p53 |
| EIF2C2/  Ago 2 | miR-125a/b*  miR-183*  miR-184  miR-27a/b  miR-99a  miR-100 | 92-93  89  87  73-74  83-84  90 | Negatively regulates p53 expression  p53  p53  p53  p53  Suppressed by p53 expression |
| EIF2C3/  Ago 3 | miR-30c*  let-7  miR-25  miR-32  miR-92 | 65  44-49  44  39  46 | p53  p53/p73/p63  p53  p53  p53 |
| EIF2C4/  Ago 4 | miR-15a  miR-130  miR-148  miR-34  let-7  miR-103/107 | 0.67303/89  0.49125/93/52  0.76735/96  96  60-65  84 | p53  p73  p53  p53  p53/p73/p63  p53 |
| KHSRP | miR-27a/b  miR-23a/b  miR-206  miR-181 | 83  -  Too close to ORF | p53  p53  p53  p53 |
| ILF3 | miR-181b*  miR-181c* | 81  82 | p53  p53 |
| ILF2 | miR-25 | 0.09432 | p53 |
| TNRC6A | miR-148 a/b  miR-23a/b  miR-30b  miR-30c*  miR-26a/b  miR-182  miR-7  miR-20b  miR-17  miR-9 | 94  97  82  61  97  59  86  *23.30*  *23.22*  *21.39* | p53  p53  p53  p53  p53  p53  p53  p53  p53  p53 |
| TNRC6B | miR-26a/b  miR-30c  miR-30b  miR-20a  miR-106b  miR-103/107  miR-200a  miR-195  miR-15/16  miR-124 | 0.09432  *58.30*  *58.39*  *39.06*  *38.96*  *37.92*  *33.05*  *32.60*  *32.56*  *32.36* | p53  p53  p53  p53  p53  p53  p53  p53  p53/p73/p63  p53 |
| TNRC6C | miR-30b  miR-30c*  miR-106  miR-26  miR-148b  miR-20b  miR-17 | 0.41443  0.41443  0.41443  *23.06*  *17.27*  *7.45*  *7.54* | p53  p53  p53  p53  p53  p53  p53 |
| P2P-R | miR-15*  miR-16*  miR-29a/b/c  miR-195 | 92  92  0.09432  92 | p53  p53  p53  p53 |
| RCK | miR-124  miR-148  miR-128  miR-143  miR-203 | 96  82  69  46  83 | p53  p53  p53  p53  p53/p73/p63 |
| GEMIN3 | miR-132 | 0.09432 | p53 |
| RAN | miR-181b/c*  miR-203 | 0.25860  41 | p53  p53/p73/p63 |
| Lin-28B | miR-181c  let-7a/c  miR-30b  miR-132  miR-203  miR-148a/b  miR-27a/b  miR-9  miR-196b  miR-124  miR-200b/c  miR-20b  miR-17 | 75  98  92  93  77  78  96  *58.02*  *18.22*  *15.63*  *12.10*  *11.70*  *11.0* | p53  p53/p73/p63  p53  p53  p53  p53  p53  p53  p53  p53  p53  p53  p53 |
| Lin-28 | miR-181c  miR-30c  let-7a/c  miR-196a/b  miR-9  mi-92a/b  miR-103/107  miR-128a/b | 92  60  93  60  93  *12.12*  *10.45*  *9.75* | p53  p53  p53/p73/p63  p53  p53  p53  p53  p53 |
| Tutase4 | miR-96  miR-132 | 99  92 | p53  p53 |
| mLIN-41 | miR-181  let-7 | 95  97 | p53/p73  p53/p73/p63 |

* Experimentally verified transcriptional targets of p53.
