# Supplementary material for: The Tumor Suppressors p53, p63, and p73 Are Regulators of MicroRNA Processing Complex
Source: PLoS One. 2010 May 12;5(5):e10615. doi: 10.1371/journal.pone.0010615 (PMC2868896; doi:10.1371/journal.pone.0010615)
Supplement: Table S3 — p53-miRs that target more than one component of the miRNA processing machinery. (0.05 MB DOC) [file pone.0010615.s003.doc]

**Supplemental Table 4**

| Ago1  TNRC6A  RCK  Lin-28B  TNRC6C  Ago 4 | miR-148a/b | 94  94  82  78  *17.27*  0.76735 |
| --- | --- | --- |
| Lin-28B  TNRC6C  TNRC6A  TNRC6B  Ago1  Ago 3 | miR-30a; 30c | 92  0.41443  82  *58.30*  *21.46*  65 |
| Dicer  TARBP2  EIF2C1/Ago1  Lin-28  TNRC6B  Ago 4 | miR-103/107 | 95/*33.45*  0.09432  81  *10.95*  *37.92*  84 |
| Dicer  TARBP2  P2PR  TNRC6B  Ago4 | miR-15/16/195 | 42/52  86/89  92/92  *32.56/32.56*  0.67303/0.89 |
| p68  p72  Ago1  TNRC6A  TNRC6B  TNRC6C | miR-20 | *8.24*  81-83  65  *23.30*  *39.06*  *7.45* |
| p72  Ago1  TNRC6A  TNRC6B  Lin-28 | miR-17 | 77  65  *23.22*  *7.54*  *11.0* |
| DROSHA  KHSRP  Ago2  Lin-28B  DGCR8 | miR-27 | 83  83  73-74  96  *8.25* |
| Dicer  p68/DDX5  KHSRP  Ago1 | miR-206 | 89  88  *-*  *14.6* |
| Dicer  P2PR  Exportin 5  Ago1 | miR-29 | 80  0.09432  *10.25*  *15.79* |
| p72  TNRC6C  Ago1  p68 | miR-106a/b | 77-78  0.41443  65  *12.61* |
| Dicer  p68  TNRC6B  Lin-28B | miR-200 | *7.8*  59  *33.05*  *12.10* |
| Lin-28  DGCR8  p72  TNRC6A | miR-9 | *58.62*  67  *15.59*  *21.39* |
| RCK  RAN  Lin-28B | miR-203 | 83  41  77 |
| TARBP2  Exportin 5  Ago4 | miR-34 | 0.09432 92  96 |
| Tutase4  Gemin3  Lin-28B | miR-132 | 92  0.09432  93 |
| RCK  DROSHA  Lin-28 | miR-128 | 69  40  *9.75* |
| TNRC6A  TNRC6B  TNRC6C | miR-26a/b | 97  0.09432  *23.06* |
| EIF2C2/Ago 2  TARBP2 | miR-183 | 89  0.09432 |
| TNRC6A  KHSRP | miR-23 | 97  - |
| RCK  Exportin 5 | miR-143 | 43  62 |
